# Modeling the Impact of Extracellular Vesicle Cargoes in the Diagnosis of Coronary Artery Disease

**DOI:** 10.3390/biomedicines12122682

**Published:** 2024-11-25

**Authors:** Peter McGranaghan, Éva Pallinger, Nóra Fekete, Pál Maurovich-Horvát, Zsófia Drobni, Béla Merkely, Luigi Menna, Edit I. Buzás, Hargita Hegyesi

**Affiliations:** 1 Biomarker Department, Charité—Universitätsmedizin, 10117 Berlin, Germany; 2 Department of Genetics, Cell and Immunobiology, Semmelweis University, 1085 Budapest, Hungary; 3 Medical Imaging Centre, Semmelweis University, 1085 Budapest, Hungary; 4 Heart and Vascular Center, Semmelweis University, 1085 Budapest, Hungary; 5 HUN-REN-SU Translational Extracellular Vesicle Research Group, 1085 Budapest, Hungary; 6 HCEMM-SU Extracellular Vesicle Research Group, 1085 Budapest, Hungary

**Keywords:** cardiovascular disease, circulating extracellular vesicles, flow cytometry, diagnosis, supportive biomarker, blood ectosome, exosome

## Abstract

**Objectives**: We aimed to assess the relationship among circulating extracellular vesicles (EVs), hypoxia-related proteins, and the conventional risk factors of life-threatening coronary artery disease (CAD) to find more precise novel biomarkers. **Methods**: Patients were categorized based on coronary CT angiography. Patients with a Segment Involvement Score > 5 were identified as CAD patients. Individuals with a Segment Involvement Score < 5 were considered control subjects. The characterization of EVs and analysis of the plasma concentration of growth differentiation factor-15 were performed using multicolor or bead-based flow cytometry. The plasma protein levels of glycogen phosphorylase, muscle form, clusterin, and carboxypeptidase N subunit 1 were determined using an enzyme-linked immunosorbent assay. Multiple logistic regression was used to determine the association of the biomarkers with the CAD outcome after accounting for established risk factors. The analysis was built in three steps: first, we included the basic clinical and laboratory variables (Model 1), then we integrated the plasma protein values (Model 2), and finally, we complemented it with the circulating EV pattern (Model 3). To assess the discrimination value of the models, an area under (AUC) the receiver operating curve was calculated and compared across the three models. **Results**: The area under the curve (AUC) values were 0.68, 0.77, and 0.84 in Models 1, 2, and 3, respectively. The variables with the greatest impact on the AUC values were hemoglobin (0.2 (0.16–0.26)) in Model 1, carboxypeptidase N subunit 1 (0.12 (0.09–0.14)) in Model 2, and circulating CD41+/CD61+ EVs (0.31 (0.15–0.5)) in Model 3. A correlation analysis showed a significant impact of circulating CD41+/CD61+ platelet-derived EVs (*p* = 0.03, r = −0.4176) in Model 3. **Conclusions**: Based on our results, the circulating EV profile can be used as a supportive biomarker, along with the conventional laboratory markers of CAD, and it enables a more sensitive, non-invasive diagnostic analysis of CAD.

## 1. Introduction

Coronary artery disease (CAD) is an atherosclerotic cardiovascular disease, which is the most common cause of death worldwide [1]. Genetic, environmental, and lifestyle factors are important determinants of cardiovascular diseases (CVD) [2,3,4]. The term “biomarker” (biological marker) was introduced more than 30 years ago, indicating a quantitative parameter that characterizes a given state of a disease, environmental effect, or metabolic process. This term was further developed as an indicator of normal biological and pathological processes [5]. Although many risk factors are known from large clinical studies, there has been an increase in the number of biomarker studies for CVD.

Cell-derived EVs are membrane-enclosed subcellular particles that are present in bodily fluids, including blood, urine, cerebrospinal fluid, and pericardial fluid [6,7]. Based on their biogenesis, exosomes are distinguished as being derived from endosomes, while ectosomes originate from the plasma membrane [8]. However, unless EVs are captured during their release, the biogenesis of EVs cannot be determined with certainty. Therefore, an operational nomenclature is suggested to distinguish small-sized EVs (sEVs, average diameter around 100–150 nm) and large EVs (lEVS) (between 200 and 800 nm and ≥1 micrometer) [9]. EVs carry specific membrane and cytosolic components, the so-called specific molecule cargo, and perform homeostatic and cell–cell signaling functions through their special molecular composition [10,11]. Although cargo molecules, including proteins, nucleic acids, and lipid entries, are available in a database [12,13], the correct interpretation of these data strongly depends on clinical conditions and sample preparation methods. Furthermore, the complex relationship between detectable biomarkers is not completely understood. Numerous studies have investigated the role of CVD-associated EVs and their applicability as biomarkers [14,15,16,17,18].

The therapeutic potential of EVs is rooted in their ability to mediate intercellular communication and deliver cargo to recipient cells. This is a feature that makes EVs particularly valuable in the treatment of diseases such as cancer, neurodegenerative disorders, and cardiovascular diseases [19,20,21]. In the context of cardiovascular diseases, EVs have been explored as vehicles for the delivery of therapeutic proteins or genetic material to promote cardiac repair following myocardial infarction. EVs can be engineered to carry growth factors or RNA molecules that stimulate angiogenesis and tissue regeneration. For instance, EVs derived from stem cells have been shown to promote tissue repair and reduce scar formation after myocardial injury [22]. Moreover, EVs can modulate inflammation and oxidative stress, which are key factors in cardiovascular pathogenesis [23].

In addition to EVs released by eukaryotic cells, EVs have counterparts in prokaryotes, e.g., outer membrane vesicles (OMVs) in Gram-negative bacteria. OMVs and EVs share similarities in their biogenesis, both being released through membrane budding processes. However, OMVs are typically smaller and involved in bacterial survival mechanisms, such as antibiotic resistance and immune evasion [24]. Recent research has suggested that OMVs could be utilized in therapeutic contexts [25], such as treating cancer and neurodegenerative and cardiovascular diseases; however, there are still challenges in terms of scaling up production, ensuring safe and efficient delivery, and addressing regulatory hurdles.

EVs have been analyzed for their potential as biomarkers, given their ability to carry disease-specific cargo and their involvement in intercellular signaling pathways pertinent to cardiovascular pathology [26]. The soluble biomarkers tested in this study include growth differentiation factor (GDF15), glycogen phosphorylase, muscle form (PYGM), clusterin, and carboxypeptidase N subunit 1 (CPN1). The EVs were selected based on their specific roles and diagnostic potential in coronary artery disease (CAD) pathophysiology. PYGM was included due to its relevance to hypoxia and ischemic conditions, which are pivotal in CAD pathogenesis in rats and swine [27,28]. Similarly, clusterin and CPN1 were selected based on evidence of their roles in cellular protection and tissue remodeling, which are critical in atherosclerosis and CAD [29,30,31]. GDF15 is well-documented for its involvement in stress response mechanisms within the cardiovascular system and has been recognized as a predictive marker in cardiovascular diseases, including CAD, due to its association with inflammation and hypoxia-related processes [32,33,34]. By integrating these biomarkers, we aimed to capture a comprehensive marker profile of CAD, encompassing both molecular and vesicular biomarkers that reflect key disease mechanisms, thereby enhancing the sensitivity and specificity of CAD diagnostics.

We tested the hypothesis that circulating EV patterns can provide incremental diagnostic value, in addition to clinical parameters and laboratory tests, in detecting CAD.

## 2. Materials and Methods

### 2.1. Patients and Sample Collection

Individuals who underwent coronary computed tomography angiography (CCTA) at the Heart and Vascular Center of Semmelweis University were retrospectively identified as CAD cases and healthy subjects. Cases were defined as patients with a Segment Involvement Score (SIS) of more than 5, while controls were defined as patients with an SIS of less than 5. The control (CAD negative) group was matched in age, gender, height, and body weight. Peripheral venous blood samples of 26 CAD patients and 14 healthy age-matched control subjects were collected from the median cubital vein using the Vacutainer^®^ Brand Plus ACD-A Tubes of Becton Dickinson (BD, San Jose, CA, USA). Biochemical and hematological parameters were evaluated using standardized clinical laboratory methods. Each donor signed a donor-informed consent form based on the guidelines and regulations of the Helsinki Declaration. The Ethics Committee of Scientific Research of Hungary (ETT-TUKEB 192/2015) approved this study.

### 2.2. Flow Cytometry

Flow cytometry was used for the characterization of circulating EVs (the validation of EVs using “common” EV markers and identification of the cellular origin of EVs) and the quantification of plasma GDF15. The tests were carried out using a FACSCalibur flow cytometer (BD, San Jose, CA, USA), and data were analyzed using CellQuestPro software 5.1 (BD, San Jose, CA, USA).

For the measurement of EVs, Megamix-Plus SSC (BioCytex, Marseille, France) calibration beads and 1 μm Silica Beads Fluo-Green Green (Kisker Biotech GmbH & Co; Steinfurt, Germany) were used for the optimization of cytometer settings and the definition of the “EV gate” (Supplementary Figure S1). Calibration beads were used to optimize the flow cytometer setting for the measurement of EVs. These fluorescent and unlabeled beads were used to define the size range in which LEVs can be detected. In Figure S1, the *y*-axis shows the SSC signal. Four different calibration bead populations can be separated on the *y*-axis.

The discrete populations representing calibration beads of known size are shown in Figure S1C. This size range (the FS–SS region that contains these discrete populations) has been used as the EV gate (see Figure S2A). The population of counting beads (bead gate) is outlined in red in Figure S2A.

Considering that the size range of EVs in biological fluids and the size of protein complexes may overlap, the following controls were included in the analysis: (1) buffer control; (2) the analysis of unstained samples (autofluorescence); (3) isotype control labeling; and (4) the re-measurement of the labeled samples after 0.1% TritonX-100 lysis. In the analysis, the fluorescence signal of the samples was plotted as a function of side scatter (as side scatter is proportional to size). The threshold was set relative to the isotype control signal, and Triton lysis was used to identify EVs. Only those stained “particles” (giving a fluorescence signal greater than the isotype control) that disappeared after detergent treatment were identified as EVs.

### 2.3. Identification of the Cellular Origin of EVs Using Flow Cytometry

Plasma samples were centrifuged at 800× *g* for 5 min to sediment any remaining cells. The supernatant was removed, and platelet-poor plasma (PPP) was prepared through centrifugation at 2500× *g* for 15 min at room temperature. Platelet-free plasma (PFP) was prepared from PPP samples through centrifugation at 12,500× *g* for 20 min at 16 °C (Z216 MK Microlite centrifuge, fixed angle 200.88 rotor, Hermle Labortechnik GmbH, Wehingen, Germany). PFP samples were divided into 300 µL aliquots and stored at −80 °C. Cell-free diluted plasma samples (1:100 in filtered PBS) were incubated with fluorochrome-conjugated monoclonal antibodies for 15 min at room temperature for the immunophenotyping of EVs (Supplementary Table S2). Annexin V staining was carried out in 2.5 mM Ca++ containing Annexin binding buffer. Unstained diluted plasma samples and EV-free monoclonal antibody solutions (staining control) were used for the evaluation of the fluorescence background according to the guideline of MIFlowCyt-EV [35]. A specific binding of fluorochrome-conjugated antibodies was examined using isotype control immunoglobulins. Differential detergent lysis by 0.1% Triton X-100 (Sigma-Aldrich, St Louis, MO, USA) was used to confirm the vesicular nature of events, as described by György et al. [36] (Supplementary Figure S2). The absolute number of EVs was determined by adding internal counting standard beads (Count Check Beads; Partec, Germany) to the diluted plasma samples. The absolute number of EVs was calculated using the following formula:

Absolute EV count (EVs/μL) = (detected EV events inside the EV gate − Triton X-100 resistant events)/acquired beads inside bead gate) × absolute count of count check beads in the tube × plasma dilution.

Since the volume of the sample used for the measurement was known, we calculated the circulating EV number considering the volume of the biological sample; the *Y*-axis in Figure S5 represents the number of “particles” (number of dots) identified as EVs based on the isotype control and Triton lysis results.

### 2.4. Flow Cytometry Multiplexed Bead-Based Immunoassays

Plasma GDF15 was quantified using the bead-based flow cytometry method (AimPlex^®^ assay technology AimPlex Biosciences, Inc., Pomona, CA, USA), according to the manufacturer’s instructions.

### 2.5. Enzyme-Linked Immunosorbent Assay (ELISA)

The plasma PYGM, clusterin, and CPN1 levels were determined using the ELISA immunological assay method. The PYGM concentration was measured using the Human PYGM ELISA Kit (Elabscience Biotechnology, Houston, TX, USA), and the CPN1 concentration was determined using the Human Carboxypeptidase N1 ELISA Kit of ABclonal Technology Company (Massachusetts, CA, USA). The clusterin concentration was assessed using the Human CLU/Clusterin ELISA Kit (Sigma, St Louis, MO, USA), according to the manufacturers’ instructions.

### 2.6. Statistical Analysis

Continuous variables were expressed as mean ± SD and compared using a *t*-test or Mann–Whitney U test, according to normal or non-normal distribution. Categorical variables were expressed as numbers (percentages). Comparisons among variables between the control and CAD groups were performed using Pearson’s chi-squared test (or Fisher’s exact test) for continuous variables and the Mantel–Haenszel chi-squared test for categorical and ordinal data. All continuous predictor variables were standardized to allow for direct comparison. Missing data were imputed using multiple imputations.

Logistic regression was performed to evaluate the association of the common clinical parameters, plasma protein biomarkers, and EV biomarkers, with the outcome of CAD diagnosis.

Three models were built to evaluate the incremental predictive value of the plasma proteins and EV biomarkers. The first model involved only the baseline clinical covariates (Model 1); then, the plasma protein biomarkers were integrated (Model 2); and finally, EV biomarkers were added to complete the model system (Model 3). Area under the curve (AUC) values and receiver operating characteristic (ROC) curves were calculated for each model, and the contribution of each analyte to the change in AUC value was determined.

To determine the relationships among the EV biomarkers, plasma biomarkers, and conventional clinical parameters, Pearson’s correlation coefficients were calculated. A *p* value of < 0.01 was defined as significant (2-tailed). The statistical analysis was performed using SAS software version 9.4.

## 3. Results

### 3.1. Patient Population

**Figure 1:**
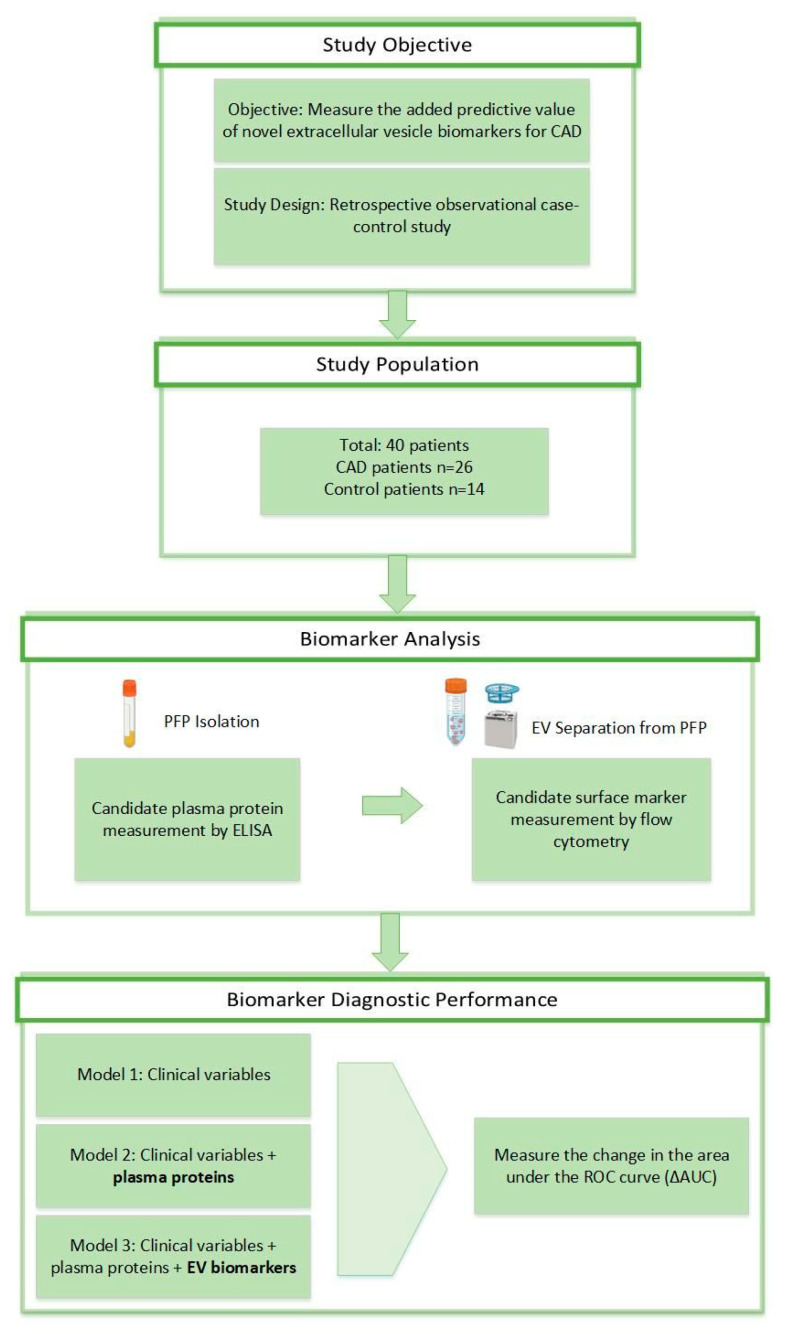
Study design. Selection of CAD and control patients. Study rationale for the prognostic biomarker study. CAD, coronary artery disease; AUC, area under the curve.

The study design is presented in Figure 1. Of the 40 total patients, 26 were diagnosed with CAD (mean age 68 ± 1.8), and 14 were healthy control patients (mean age 62 ± 2.6). Baseline characteristics of the patient population are shown in Supplementary Table S1. Height, monocytes, red blood cells, creatine kinase, lactate dehydrogenase (LDH), hemoglobin, hematocrit, and mean corpuscular hemoglobin concentration (MCHC) were significantly different between the two groups (*p* < 0.05). The coronary CT angiography (CCTA) results of cases and controls were all significantly different between the CAD and control groups (Supplemental Figure S3).

### 3.2. Circulating Plasma Proteins

**Figure 2:**
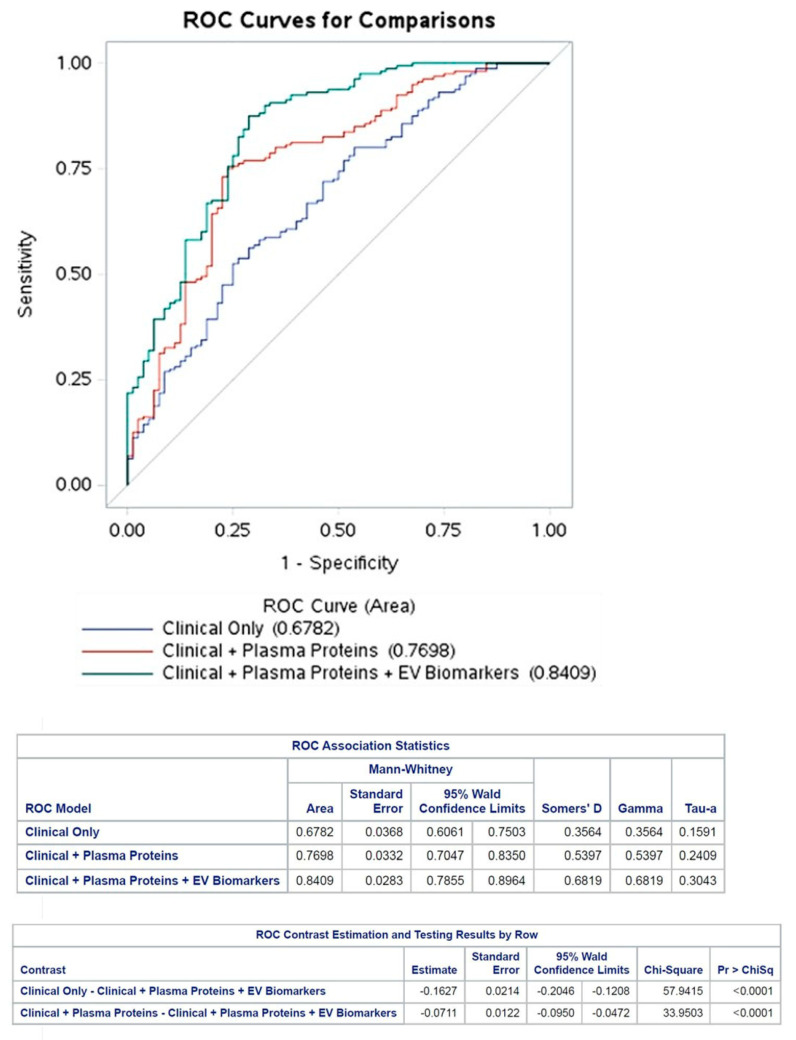
ROC curves to predict measures of CAD in the tree models. Discrimination analysis for extracellular vesicle (EV) biomarkers for predicting coronary artery disease diagnosis represented by areas under the receiver operating characteristic (ROC) curve.

Significantly elevated levels of GDF15 (CAD:1600 ± 188 pg/mL; control: 1003 ± 94.1 pg/mL; *p* = 0.039) and CPN1 (CAD: 142.5 ± 4.7 pg/mL; control: 126.2 ± 6.2 pg/mL; *p* = 0.04) could be detected in coronary artery disease patients, but the plasma concentrations of PYGM (CAD: 7740 ± 4.45 ng/mL; control: 1213 ± 0.76 ng/mL; *p* > 0.05) and clusterin (CAD: 972.4 ± 246.8 pg/mL; control: 942.4 ± 306 pg/mL; *p* > 0.05) did not differ compared to the control individuals. Although the correlation analysis showed that the concentration changes in the detected plasma proteins were independent, the ROC analysis of GDF15 and CPN1 (Figure 2) validated that these parameters can distinguish the two diagnostic groups. According to the correlation matrix analysis, the investigated proteins were identified as independent variables. The distribution of the measured plasma proteins in control and CAD patients is illustrated in Supplemental Figure S4.

### 3.3. Circulating EV Pattern in Human Plasma Samples

Platelet-derived EVs were specified by CD41 (alpha IIb integrin, platelet GPIIb) and CD61 (platelet glycoprotein GPIIIa), while activated platelet-derived EVs were determined by the CD62P (P-selectin/GMP-140/PADGEM) expressions. EVs secreted by endothelial cells were identified by the exofacial presence of CD31 (platelet endothelial cell adhesion molecule/PECAM-1). CD14 (co-receptor for bacterial lipopolysaccharide) was used for the classification of monocyte-derived EVs, and CD142 (coagulation factor III/thromboplastin) was used for the analysis of tissue factor-expressing procoagulant vesicles. CRP-associated circulating EVs were also measured. Fluorochrome-labeled annexin V binding was used as a “common EV marker”, although its positivity also reflects activation in the case of platelet-derived EVs [37]. The distribution of the EV biomarkers in control and CAD patients is illustrated in Supplemental Figure S5.

An ROC analysis was performed to assess the incremental diagnostic value of the selected plasma proteins and EV biomarkers. The AUC values were used to analyze the diagnostic accuracy of the tests, i.e., how well the detected parameters differentiated the patient groups [38]. The AUC values were 0.68 in Model 1, 0.77 in Model 2, and 0.84 in Model 3 (Figure 2). The variables with the greatest impact on the AUC values were hemoglobin (0.2 (0.16–0.26)) in Model 1, CPN1 (0.12 (0.09–0.14)) in Model 2, and circulating CD41+/CD61+ EVs (0.31 (0.15–0.5)) in Model 3. (Supplemental Figure S6).

## 4. Discussion

In the present study, we characterized the circulating EV pattern of patients with CAD from the perspective of cells involved in the pathomechanism of the disease, with vesicular markers of hypoxia-reperfusion injury. Specifically, PYGM, clusterin, CPN1, and EVs were selected as biomarkers based on their distinct functions and potential diagnostic relevance in the pathophysiology of CAD [26,27,28,29,30,31]. The proteins PYGM and clusterin are increasingly recognized as soluble cardiovascular biomarkers [27,30]. PYGM, which is primarily linked to muscle energy metabolism, has been associated with ischemic conditions and metabolic dysfunction, potentially indicating cardiovascular risk. Clusterin, which is a glycoprotein involved in cellular protection, is found to be elevated in response to vascular injury and atherosclerosis. Both proteins’ roles in inflammatory and metabolic pathways contribute to their value as biomarkers for cardiovascular health, as they can signal early disease onset and progression [29]. The clinical relevance of GDF15 has been confirmed by numerous earlier studies [32,33,34]; therefore, we also investigated its concentration in our patient groups.

We hypothesized that complementing conventional CAD risk factors with plasma protein markers and the circulating EV pattern can result in a more sensitive prediction system. The diagnosis of CAD is usually based on imaging in combination with laboratory testing. It would be beneficial if CAD could be detected before imaging studies are performed and attenuated prior to irreversible complications or death. This could be accomplished through a robust biomarker panel to detect CAD. Traditional serum biomarkers are valuable because they allow for the earlier diagnosis of the disease and can be measured relatively easily [39].

Several working groups are looking for potential EV-associated biomarkers in CVD [40,41]. A recent study used multicolor flow cytometry for the identification of the cellular origin of circulating EVs [42]. In our study, we compared the circulating EV patterns of CVD patients and healthy controls to detect markers capable of distinguishing individuals in each group. We found that the absolute number of circulating CD142 + and CD31 + EVs could clearly distinguish the CAD patients from the control individuals. Significantly elevated levels of CPN1 could be detected in coronary artery disease patients, and the ROC analysis of CPN1 showed that this parameter can also distinguish the two diagnostic groups.

This study has some limitations. First, this was a cross-sectional study that had no follow-up; thus, we could not clearly establish a causal relationship between biomarkers and the development of CAD. Second, this study only included Hungarian subjects; therefore, our results may not fully apply to populations in different geographical regions. Third, the sample size was limited, and larger studies are required. Future studies will address these limitations by incorporating larger, more diverse cohorts and exploring standardized methodologies for EV analysis.

Based on our results, circulating EV profiles can be used as an additional biomarker, along with the conventional laboratory markers of CAD, which may enable a more sensitive, non-invasive diagnosis of CAD. In addition to their diagnostic significance, our results contribute to a better understanding of both the pathophysiology and biological mechanisms of CAD.

## 5. Conclusions

Based on our results, we recommend integrating the detection of CRP-associated, circulating platelet-derived, endothelial cell-derived, and monocyte-derived EVs, along with plasma CPN1 concentrations, into the CAD diagnostic/prognostic panel as supportive biomarkers.

## Data Availability

The original contributions presented in the study are included in the article/supplementary material, further inquiries can be directed to the corresponding author.

## Supplementary Materials

The following supporting information can be downloaded at https://www.mdpi.com/article/10.3390/biomedicines12122682/s1, Table S1: Clinical parameters of patients; Table S2: List of antibodies and dyes used for immunofluorescence studies; Figure S1: Instrument settings; Figure S2: Flow cytometric analysis of EVs according to MIFlowCyt guidelines; Figure S3: CTTA parameters of control and CAD groups; Figure S4: Plasma proteins in cases and controls; Figure S5: EVs in the blood of cases and controls; Figure S6: Feature importance ranking.
