# Supplementary material for: Modeling the Impact of Extracellular Vesicle Cargoes in the Diagnosis of Coronary Artery Disease
Source: Biomedicines. 2024 Nov 25;12(12):2682. doi: 10.3390/biomedicines12122682 (PMC11727391; doi:10.3390/biomedicines12122682)
Supplement: Supplementary file 1 [file biomedicines-12-02682-s001.zip › Figure S1. Instument settings.pdf]

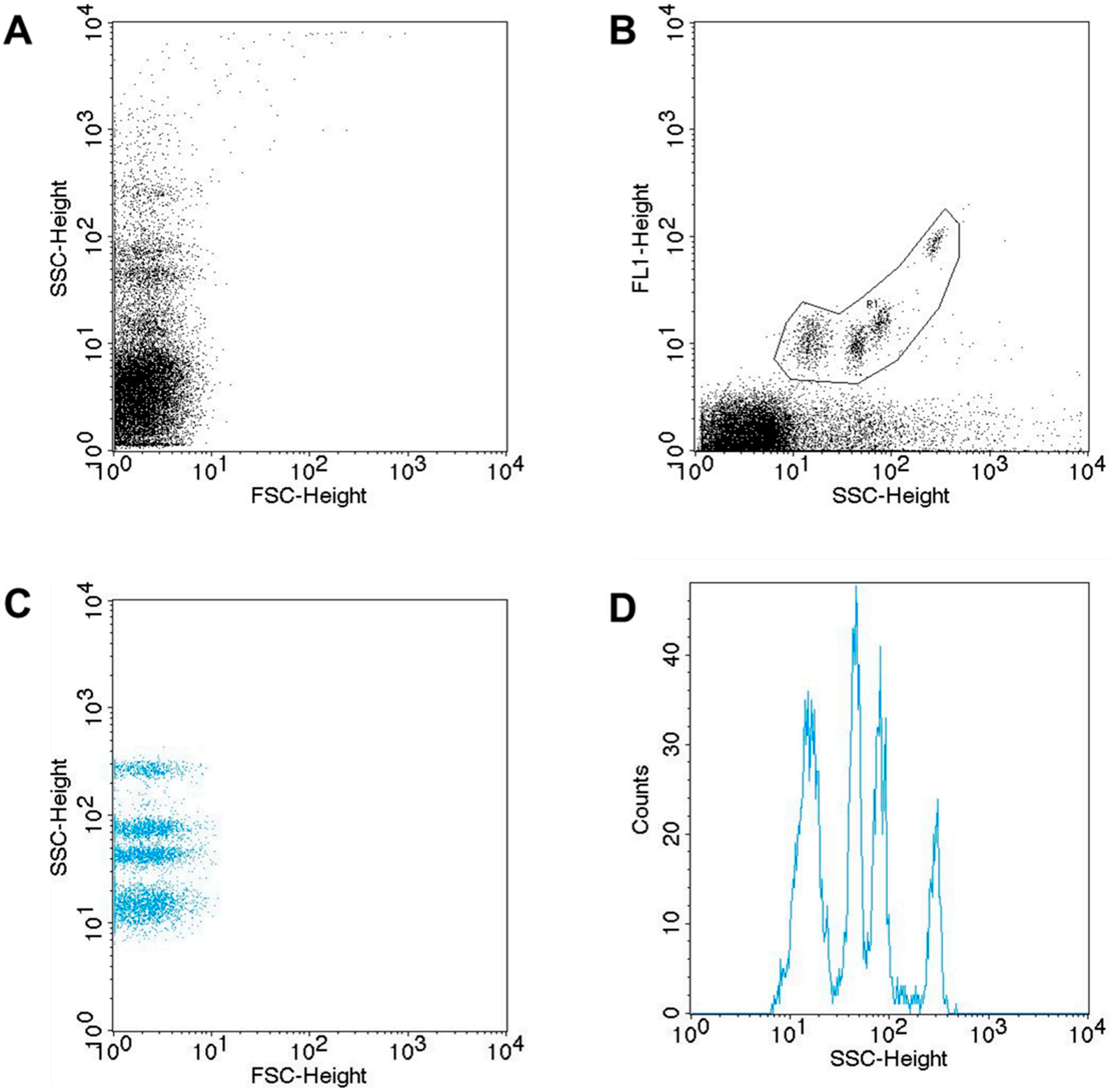

Figure S1. Instrument settings. The instrument settings and gates were defined by Megamix-Plus SSC beads (Biocytex, France) (A to D) and were optimized with 1 µm Silica Beads Fluo-Green Green (Kisker Biotech GmbH & Co; Steinfurt, Germany) The EV gate defined by these calibration beads was analyzed. A) Representative dot plot shows the forward scatter (FSC) / side scatter (SSC) picture of diluted Megamix-Plus SSC beads. B) Representative dot plot shows the green fluorescence signal (FL1) of Megamix-Plus SSC beads compared to the side scatter (SSC). FL1 signal was used for the definition of beads by gating (R1 gate). C) FSC / SSC dot plot shows the bead populations inside the R1 gate defined on the basis of FL1 fluorescence. D) Megamix beads of different sizes can be clearly distinguished on an SSC histogram.
