# Supplementary material for: Modeling the Impact of Extracellular Vesicle Cargoes in the Diagnosis of Coronary Artery Disease
Source: Biomedicines. 2024 Nov 25;12(12):2682. doi: 10.3390/biomedicines12122682 (PMC11727391; doi:10.3390/biomedicines12122682)
Supplement: Supplementary file 1 [file biomedicines-12-02682-s001.zip › Figure S2. Flow cytometric analysis of EVs according to MIFlowCyt guidelines.pdf]

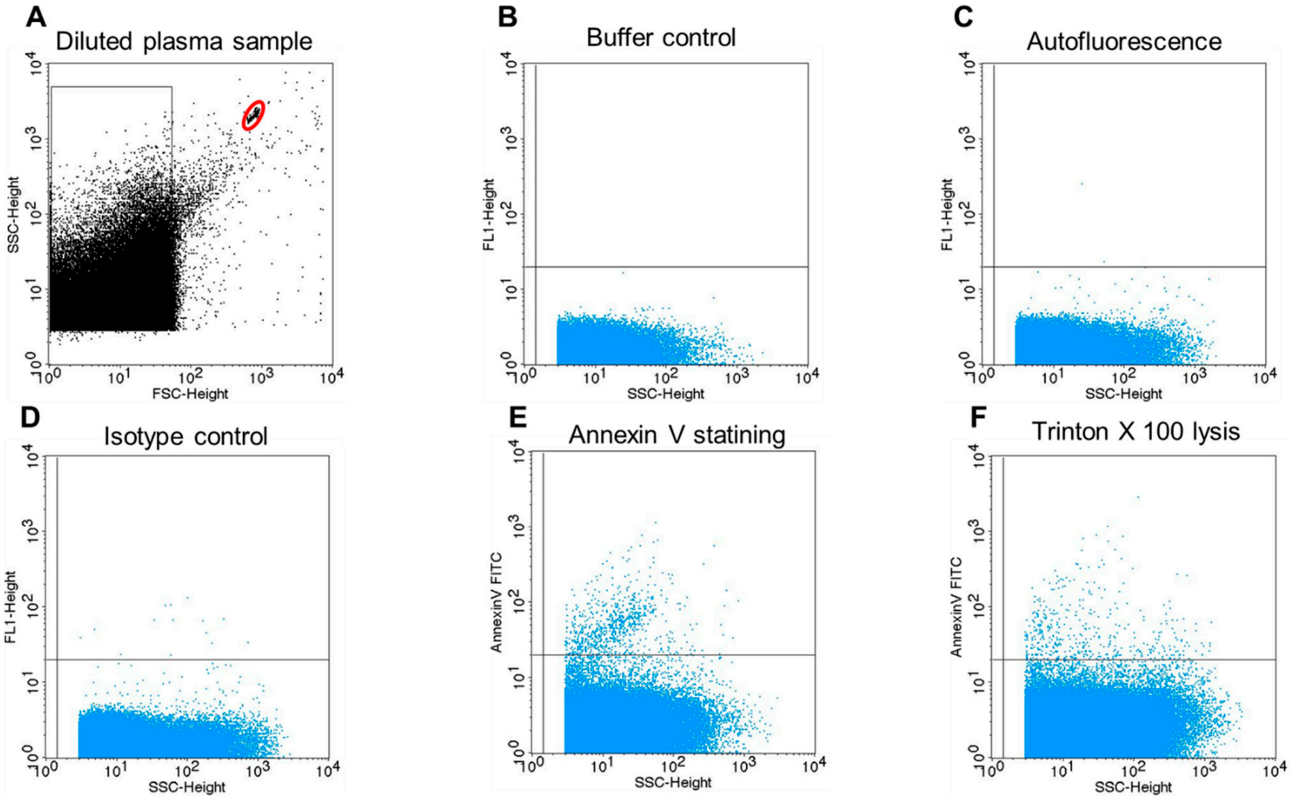

Figure S2. Flow cytometric analysis of EVs according to MIFlowCyt guidelines A) Representative dot plot shows the FSC / SSC picture of a diluted plasma sample. R1 gate represents EV gate defined by the using of calibration beads (Megamix-Plus SSC and silica beads). R2 gate (red circle) define the gate of Count Check beads which were used for absolute counting (see in Materials and Methods section). B) Buffer control was prepared by mixing filtered annexin binding buffer and FITC-conjugated AnnexinV. Representative dot plot shows the FL1 fluorescence signal inside the EV gate, which was used for determining background fluorescence signal. C) Autofluorescence means the fluorescence signal of unstained EVs inside EV gate. D) Isotype control was prepared by using of isotype control antibodies, which can only aspecifically bind to the EVs. E) Representative dot plot shows the FL1 fluorescence signal of EVs labelled by FITC-conjugated Annexin V. F) Triton X 100 detergent lysis was used for the evaluation of vesicular nature of detected events (see in Materials and Methods section). B, C, D and F dot plots were applied for gating quantitative measurements.
