# Supplementary material for: Modeling the Impact of Extracellular Vesicle Cargoes in the Diagnosis of Coronary Artery Disease
Source: Biomedicines. 2024 Nov 25;12(12):2682. doi: 10.3390/biomedicines12122682 (PMC11727391; doi:10.3390/biomedicines12122682)
Supplement: Supplementary file 1 [file biomedicines-12-02682-s001.zip › Figure S3. CTTA parameters of control and CAD groups.pdf]

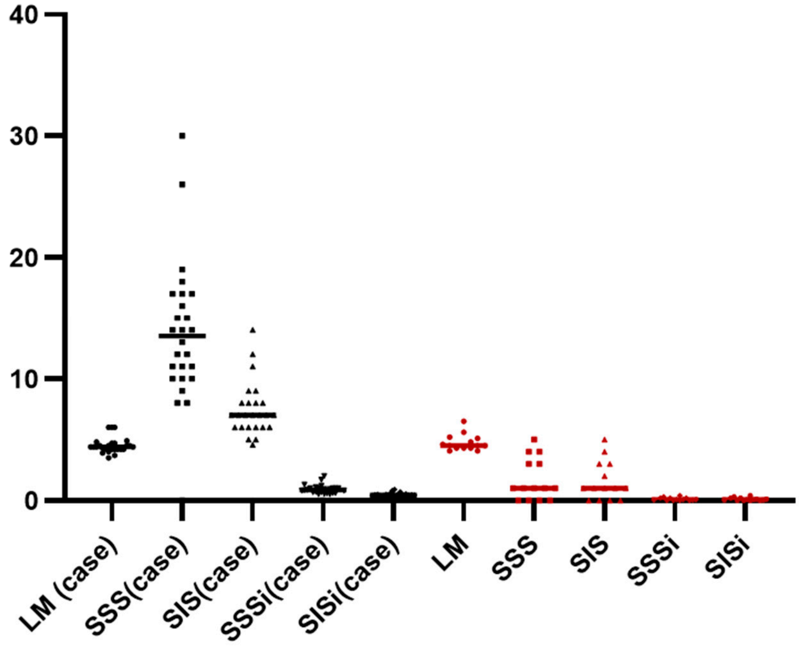

Figure S3. CTTA parameters of control and CAD groups. Left main coronary artery (LM), Segment stenosis score (SSS), Segment involvement score (SIS), (SSSi) = SSS / number of segments, Segment involvement score index (SISi).
