# Supplementary material for: Modeling the Impact of Extracellular Vesicle Cargoes in the Diagnosis of Coronary Artery Disease
Source: Biomedicines. 2024 Nov 25;12(12):2682. doi: 10.3390/biomedicines12122682 (PMC11727391; doi:10.3390/biomedicines12122682)
Supplement: Supplementary file 1 [file biomedicines-12-02682-s001.zip › Figure S4. Plasma proteins in cases and controls.pdf]

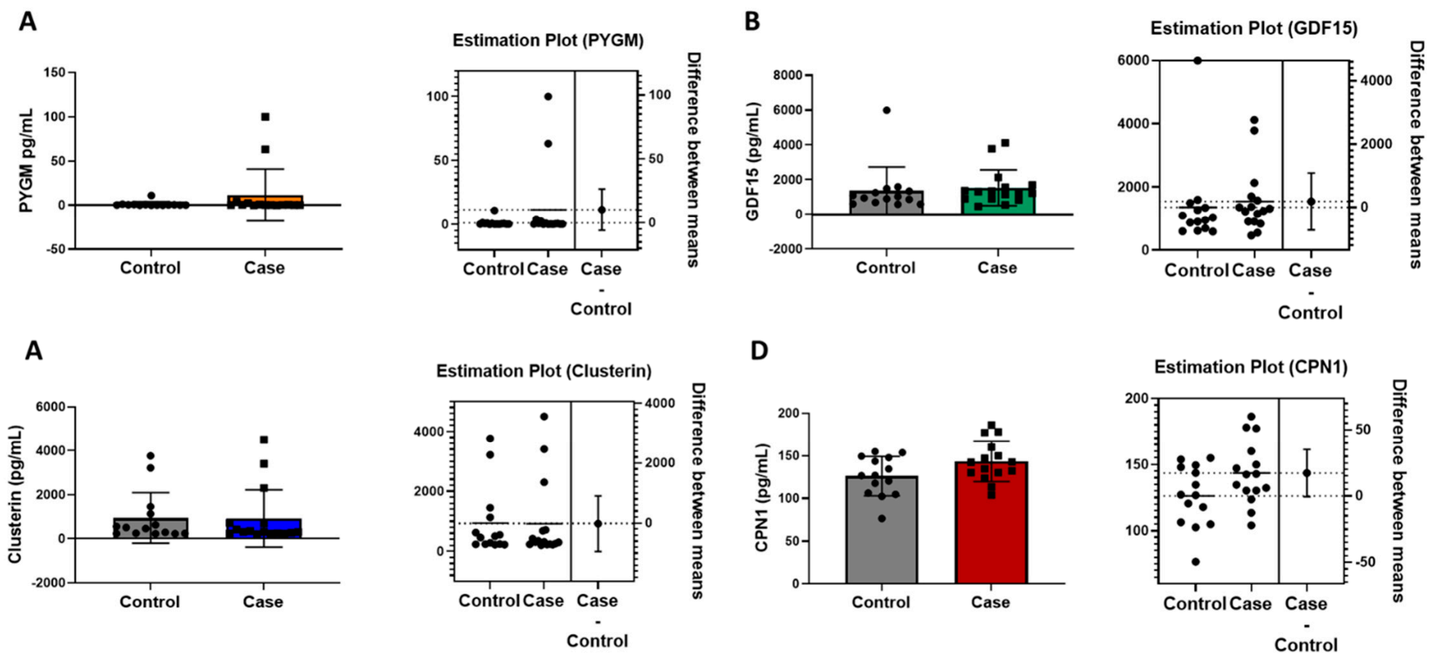

Figure S4. Plasma proteins in cases and controls. Quantification of the concentration of the PYGM, Clusterin, GDF-15 and CPN1 in the case and the control groups. Estimation plots were made with GraphPad 8.0 software.
