# Supplementary material for: Modeling the Impact of Extracellular Vesicle Cargoes in the Diagnosis of Coronary Artery Disease
Source: Biomedicines. 2024 Nov 25;12(12):2682. doi: 10.3390/biomedicines12122682 (PMC11727391; doi:10.3390/biomedicines12122682)
Supplement: Supplementary file 1 [file biomedicines-12-02682-s001.zip › Figure S5. EVs in the blood of cases and controls_rev.pdf]

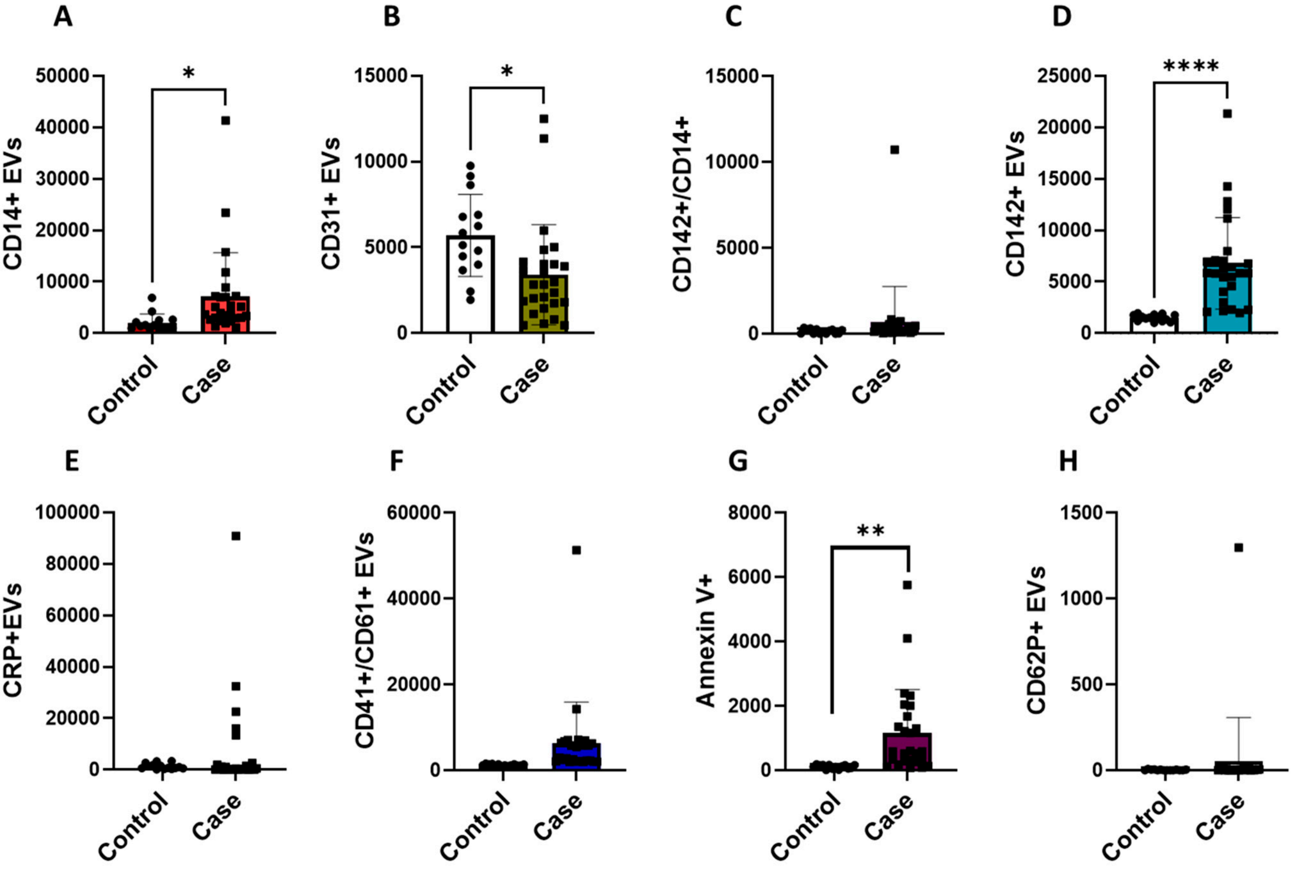

Figure S5. EVs in the blood of cases and controls. Cellular origin of EVs in samples, demonstrating that the large majority of EVs released during recirculation are platelet-derived (platelet tissue factor (TF) as CD142+) and monocyte-derived (CD14+). Statistically significant differences (*=p < 0.05, **=p<0.01, ****=p<0.001) are marked by asterisks.
