# Supplementary material for: Modeling the Impact of Extracellular Vesicle Cargoes in the Diagnosis of Coronary Artery Disease
Source: Biomedicines. 2024 Nov 25;12(12):2682. doi: 10.3390/biomedicines12122682 (PMC11727391; doi:10.3390/biomedicines12122682)
Supplement: Supplementary file 1 [file biomedicines-12-02682-s001.zip › Figure S6. Feature importance ranking.pdf]

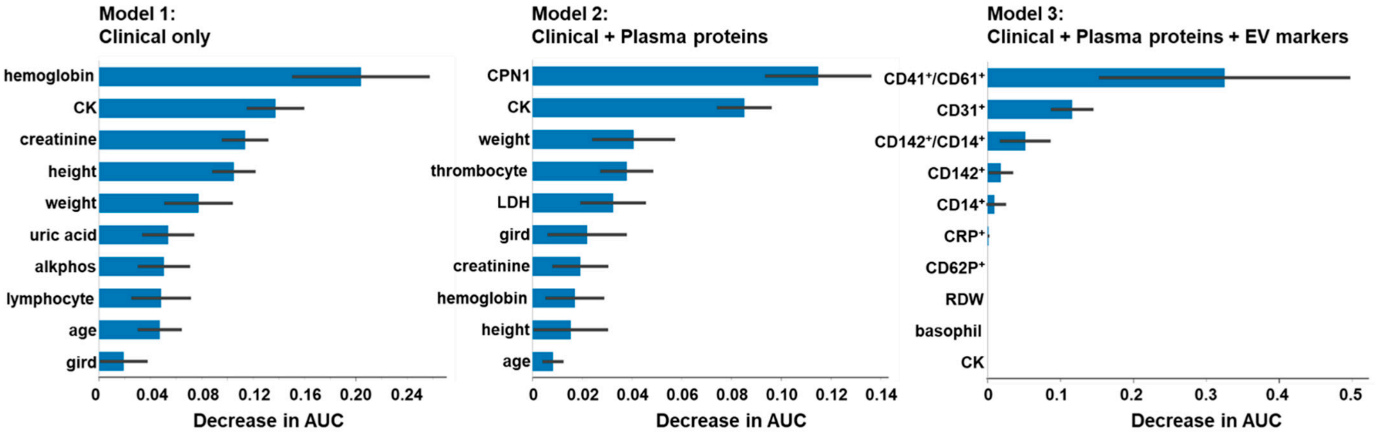

Figure S6. Feature importance ranking. List of the relative importance of Model 1, Model 2 and Model 3 variables in the machine learning-based model for the evaluation of CAD.
