# Supplementary material for: Modeling the Impact of Extracellular Vesicle Cargoes in the Diagnosis of Coronary Artery Disease
Source: Biomedicines. 2024 Nov 25;12(12):2682. doi: 10.3390/biomedicines12122682 (PMC11727391; doi:10.3390/biomedicines12122682)
Supplement: Supplementary file 1 [file biomedicines-12-02682-s001.zip › Table S1. Clinical parameters of patients.pdf]

Supplementary Materials for

Modeling the Impact of Extracellular Vesicle Cargoes in the Diagnosis of Coronary Artery Disease

Peter McGranaghan, Éva Pallinger, Nóra Fekete, Pál Maurovich-Horvát, Zsófia Drobni,

Béla Merkely, Luigi Menna and Edit I. Buzás * and Hargita Hegyesi *

* Correspondence: buzas.edit@semmelweis.hu (E.I.B.); hegyesi.hargita@semmelweis.hu (H.H.); Tel.: +36-20595-0942 (H.H.)

Clinical parameters of patients

	Case (CAD) n=26	Control n=14	p value
**age**	68.44±1.85	62.29±2.68	>0.05
**male n (%)**	17 (65.38)	12 (85,71)	>0.05
**weight**	84.86± 3.54	91.65±5.31	>0.05
**height**	166.84± 2.37	175.57±2.37	>0.05
**waist circumference**	104.83± 2.08	110.85±5.73	>0.05
**Comorbidities**			
**Hypertension n (%)**	21 (80.77)	7 (50)	
**Diabetes n (%)**	5 (19.23)	2 (14.28)	
**Dislipidemia n (%)**	16 (61.54)	2 (14.28)	
**Smoking (yes/no)**	13 (50)	5 (35.72)	
**Myocardial infarction (yes/no)**	1 (3.85)	0 (0)	
**Laboratory data**			
**WBC ($x10^6$/L)**	6.8±1.45	6.41±1.25	>0.05
**Neutrophil**	61.62±7.8	60.66±6.32	>0.05
**Lymphocyte**	28.65±6.82	28.64±4.9	>0.05
**Monocyte**	6.61±1.52	7.69±1.47	**0,04**
**Eosinphil**	2.87±1.26	2.64±1.48	>0.05
**RBC**	4.69±0.45	5.06±0.35	**0,01**
**Basophil**	0.29±0.17	0.36±0.14	>0.05
**CK**	109.44±55.03	201.79±118.51	**0,01**
**CKMB**	14.38±5.28	15.84±3.34	>0.05
**LDH**	294.14±50.43	324.36±36.36	**0,04**
**RDW**	13.64±0.86	13.51±0.69	>0.05
**Thrombocyte**	219.72±49.09	217.36±62.86	>0.05
**Creatinine**	79.34±11.5	75.93±15.92	>0.05
**Urea**	5.97±1.31	5.58±1.06	>0.05
**Glucose**	6.27±1.53	6.07±1.34	>0.05
**T-bilirubin**	9.48±3.33	12.75±6.49	>0.05
**Cholesterol**	5.0±1.37	5.06±1.33	>0.05
**Triglyceride**	2.08±2.25	1.37±0.82	>0.05
**HDL**	1.54±0.45	1.5±0.44	>0.05
**LDL**	2.87±1.16	3.28±1.22	>0.05
**Hemoglobin**	140.43±13.38	150.86±9.19	**0,01**
**Ht**	0.41±0.04	0.44±0.03	**0,03**
**MCV**	88.91±4.1	87.1±5.67	>0.05
**MCH**	30.±1.49	29.88±1.59	>0.05
**MCHC**	337.61±8.3	343.43±7.25	**0,03**
**Na**	139.49±1.72	140.36±1.98	>0.05
**K**	4.44±0.33	4.44±0.57	>0.05
**Cl**	101.26±1.51	102.03±2.18	>0.05
**GFR**	74.93±8.39	82.79±8.75	**0,01**
**Albumin**	46.64±1.89	47.68±2.1	>0.05
**Ca**	2.41±0.13	2.32±0.11	**0,02**
**Mg**	0.87±0.06	0.87±0.09	>0.05
**Protein**	70.48±3.58	69.62±3.95	>0.05
**CRP**	3.09±3.0	1.76±1.24	>0.05
**Uric acid**	333.74±72.95	341.46±88.36	>0.05
**GOT**	20.96±5.14	26.93±9.73	**0,05**
**GPT**	21.6±7.82	29.86±13.42	**0,05**
**alkphos**	76.29±26.35	72.29±12.8	>0.05
**amylase**	70.37±26.15	70.64±18.28	>0.05
**GGT**	24.72±14.1	30.71±19.93	>0.05
**phosphate**	1.07±0.14	1.02±0.12	>0.05

Table S1. Baseline characteristics of patients. WBC creatine kinase (CK), creatine kinase-MB (CK-MB), lactic dehydrogenase (LDH), red blood cell distribution width (RDW), mean corpuscular volume (MCV) Statistically significant value are given as bold.
