# Supplementary material for: Modeling the Impact of Extracellular Vesicle Cargoes in the Diagnosis of Coronary Artery Disease
Source: Biomedicines. 2024 Nov 25;12(12):2682. doi: 10.3390/biomedicines12122682 (PMC11727391; doi:10.3390/biomedicines12122682)
Supplement: Supplementary file 1 [file biomedicines-12-02682-s001.zip › Table S2. List of antibodies and dyes used for immunofluorescence studies.pdf]

List of antibodies and dyes used for immunofluorescence studies

Antibody/Dye name	Clone*	Manufacturer	Catalog number	Detected cellular origin
FITC Annexin V		Biolegend	640906	LEV
FITC anti-human CD81	M38	Molecular P.	A15753	LEV
PE anti-human CD63	MEM 259	Sigma	SAB4700218	LEV
PerCP anti-human CD9	M-L13	BD	561329	LEV
FITC anti-human CD41a	HIP8	BD	555466	platelet-derived – LEV
APC anti-human CD62P	AK-4	BD	555523	activated platelet-derived – LEV
PE anti-human CD142	NY2	SONY	2426015	tissue factor expressing – LEV
FITC anti-human CD14	61D3	eBioscience	11014942	monocyte-derived - LEV
Alexa-647 anti-human CRP	polyclona l	Bioss	bs-0155R-A647	CRP-bound - LEV
PE anti-human CD31	WM59	BioLegend	303106	endothelial cell-derived LEV

Table S2. List of primary antibodies used for the measurement of flow-cytometry
